# Genetic dissection of the Arabidopsis spaceflight transcriptome: Are some responses dispensable for the physiological adaptation of plants to spaceflight?

**DOI:** 10.1371/journal.pone.0180186

**Published:** 2017-06-29

**Authors:** Anna-Lisa Paul, Natasha J. Sng, Agata K. Zupanska, Aparna Krishnamurthy, Eric R. Schultz, Robert J. Ferl

**Affiliations:** 1 Department of Horticultural Sciences, University of Florida, Gainesville, Florida, United States of America; 2 Interdisciplinary Center for Biotechnology and Research, University of Florida, Gainesville, Florida, United States of America; Institute of Genetics and Developmental Biology Chinese Academy of Sciences, CHINA

## Abstract

Experimentation on the International Space Station has reached the stage where repeated and nuanced transcriptome studies are beginning to illuminate the structural and metabolic differences between plants grown in space compared to plants on the Earth. Genes that are important in establishing the spaceflight responses are being identified, their roles in spaceflight physiological adaptation are increasingly understood, and the fact that different genotypes adapt differently is recognized. However, the basic question of whether these spaceflight responses are actually required for survival has yet to be posed, and the fundamental notion that spaceflight responses may be non-adaptive has yet to be explored. Therefore the experiments presented here were designed to ask if portions of the plant spaceflight response can be genetically removed without causing loss of spaceflight survival and without causing increased stress responses. The CARA experiment compared the spaceflight transcriptome responses in the root tips of two Arabidopsis ecotypes, Col-0 and WS, as well as that of a PhyD mutant of Col-0. When grown with the ambient light of the ISS, *phyD* plants displayed a significantly reduced spaceflight transcriptome response compared to Col-0, suggesting that altering the activity of a single gene can actually improve spaceflight adaptation by reducing the transcriptome cost of physiological adaptation. The WS genotype showed an even simpler spaceflight transcriptome response in the ambient light of the ISS, more broadly indicating that the plant genotype can be manipulated to reduce the cost of spaceflight adaptation, as measured by transcriptional response. These differential genotypic responses suggest that genetic manipulation could further reduce, or perhaps eliminate the metabolic cost of spaceflight adaptation. When plants were germinated and then left in the dark on the ISS, the WS genotype actually mounted a larger transcriptome response than Col-0, suggesting that the in-space light environment affects physiological adaptation, which implies that manipulating the local habitat can also substantially impact the metabolic cost of spaceflight adaptation.

## Introduction

Plants, as much as any other complex eukaryotic organisms, mount specific transcriptional responses to an environment in order to accomplish physiological adaptation and survival in that environment. Heat shock, salt, cold, drought and hypoxia are among the most well studied environmental responses in plants, and mutant studies of these responses have been key to showing that a given aspect of the response is necessary and appropriate for survival (e.g. [1, 2–8]). However, in each of these examples, the environmental stress was a part of the evolutionary history of plants and the development of a stress response for survival seems logically appropriate. Knocking out those appropriate responses has a negative effect on survival under that environmental stress (e.g. [9, 10, 11]), and manipulation of the response can modify the degree of stress that can be overcome (e.g. [12, 13–15]). Spaceflight, however, presents an environmental situation that is well outside of the evolutionary history of plants, wherein plants have had no exposure to develop adaptive strategies, and this situation creates the possibility that environmental signals in spaceflight could be inappropriately activating non-adaptive responses.

The possibility of non-adaptive responses to spaceflight presents an added dimension to the goal of understanding plant growth in space and the fundamental biological questions of adaptation to truly novel environments. What is the measure of successful adaptation to a new environment? Is it possible to gauge the impact of a novel environment on the basis of molecular responses? Can inappropriate responses be genetically removed, thereby reducing the transcriptome costs of developing appropriate adaptation responses? In this study, these questions were explored simply on the basis of measuring the intensity and complexity of the patterns of differential gene expression in response to spaceflight in different genotypes. The data presented here look at a very specific set of cells–the last 2 mm of the primary root of Arabidopsis (*Arabidopsis thaliana*), which represents the primary gravity-sensing region of the plant root (e.g. [16, 17]. These data compared specific genotypes in order to dissect the spaceflight responses of plants as they germinated and continued their development on the International Space Station. These plants were not undergoing transitional physiological adaptation to a new environment, but rather, from the moment of germination, they experienced an environment that is outside the evolutionary experience of any terrestrial organism.

This study therefore identifies the genes engaged by the cells of the root tip in the spaceflight environment compared to similar plants on the ground, and then asks how variations in genetic background, and the lighting sub-environment on the International Space Station, impact the differential expression of genes.

The experimental design extrapolates primarily from the APEX01 (Advanced Plant Experiment 01) Arabidopsis spaceflight experiment. APEX01 illustrated that during spaceflight in microgravity, Arabidopsis plants utilized the gradient of light established in the growth hardware to orient the directional axis of growth, and demonstrated unique, genotype-specific patterns of root growth [18, 19]. On the ground, the roots grew vertically down the surface of the plate, but surprisingly, so did the roots on orbit, but not straight “down” in a negatively phototropic direction; the roots instead progressed in a distinctive skewing pattern, such as seen on Earth when grown on tilted surfaces (e.g. [20]. The skewing growth pattern was interesting from the perspective that skewing was long thought to require gravity, but also from the perspective that the two genotypes flown–Wassilewskija (WS) and Columbia-0 (Col-0)–exhibited distinctly different skewing morphometrics, which were enhanced over what was typical on the ground. WS and Col-0 are genotypically distinct and therefore possess potentially different tools to respond to any given set of environments [21, 22]. Spaceflight studies by Blancaflor and colleagues demonstrated that Col-0 skews more dramatically in the dark than in the light, and that genes associated with the actin cytoskeleton can impact root morphology in microgravity [23, 24]. Spaceflight studies by Kiss and colleagues established that the interplay of specific wavelengths of light and manipulation of light-receptor genes can have a dramatic impact on the morphology of seedling growth in microgravity and fractional gravity, and have shown that the Arabidopsis cultivar Landsberg also exhibits skewing [25–29]. Light plays a larger role in root behavior in the spaceflight environment, and nuanced impact of the genomic background appears to be more easily revealed when gravity is not the dominant tropic force acting on the plant.

Among the genes that differ between WS and Col-0 is phytochrome D (PhyD), which encodes a PHYB-like member of the family. PHYD appears to work in conjunction with PHYB for shade avoidance in terrestrial habitats [30, 31], but appears to have virtually no role in gravitropism [32]. However, although WS lacks PhyD transcription it does contain normal levels of PhyA, PhyB, and PhyC [30]. There is considerable overlap in the roles of the members of the phytochrome family, but a mutation in PhyD in a Col-0 background mimics some of the phenotypic features of the WS ecotype (e.g. [33]). The role of PhyD in shade avoidance, and also its lack of a role in gravity sensing, contributed to our interest in the gene, but the fact that the deletion of a single, seemingly inconsequential gene in Col-0 could also phenocopy features of the wild-type WS cultivar was especially intriguing, and led us to choose the Col-0 *phyD* line for inclusion in a transcriptome comparison of the wild type WS and Col-0 genotypes in the spaceflight environment.

The experiment described here examined the influence of genotype on the physiological adaptation to spaceflight as measured by gene expression patterns. RNAseq transcriptome profiling provided the metric by which to measure the impact of the environment, and address whether it is possible to eliminate all or part of the spaceflight response by manipulating the genotype of the plant.

## Materials and methods

### CARA experiment seed lines and planting

Three seed lines wild-types Wassilewskija (WS), Columbia-0 (Col-0) and Col-0 PhyD (*phyD*) (SALK_027956C) were tested for viability, sterility and the ability to maintain dormancy before launch. Vetted batches of seeds were planted on Phytagel plates [18]. One genotype was planted per plate, and six plates were planted of each genotype. One complete set of 18 plates was planted for growth on the International Space Station (ISS), and a comparable set of 18 plates was planted 24 hours later for the ground controls. The plates were wrapped such that every surface of the plate was covered by at least two layers of Duvetyne Black-Out cloth (Seattle Fabrics) to help preserve dormancy [34]. The plates were stored at 4°C until launch (six days). All seed lines are available through TAIR (https://www.arabidopsis.org/).

### Launch, orbital operations of flight and ground control plates

The plates of the CARA experiment launched in a cold-stow bag to maintain the plates at 4°C within the Dragon Capsule. Launch of SpaceX CRS-3 occurred on April 18^th^, 2014 to the ISS (increment 39). The cargo Dragon Capsule was carried by a Falcon 9 rocket launched from Complex 40, Cape Canaveral Air Force Station, Florida. CARA is also identified by the NASA operations nomenclature (OpNom) of “Petri Plants”, and was one of the ARK1 payloads flown by the Center for Advancement of Science in Space (CASIS) (http://www.nasa.gov/mission_pages/station/research/experiments/1064.html). The astronaut attending the experiment on the ISS was Expedition 39 Flight Engineer Steve Swanson.

**Fig 1:**
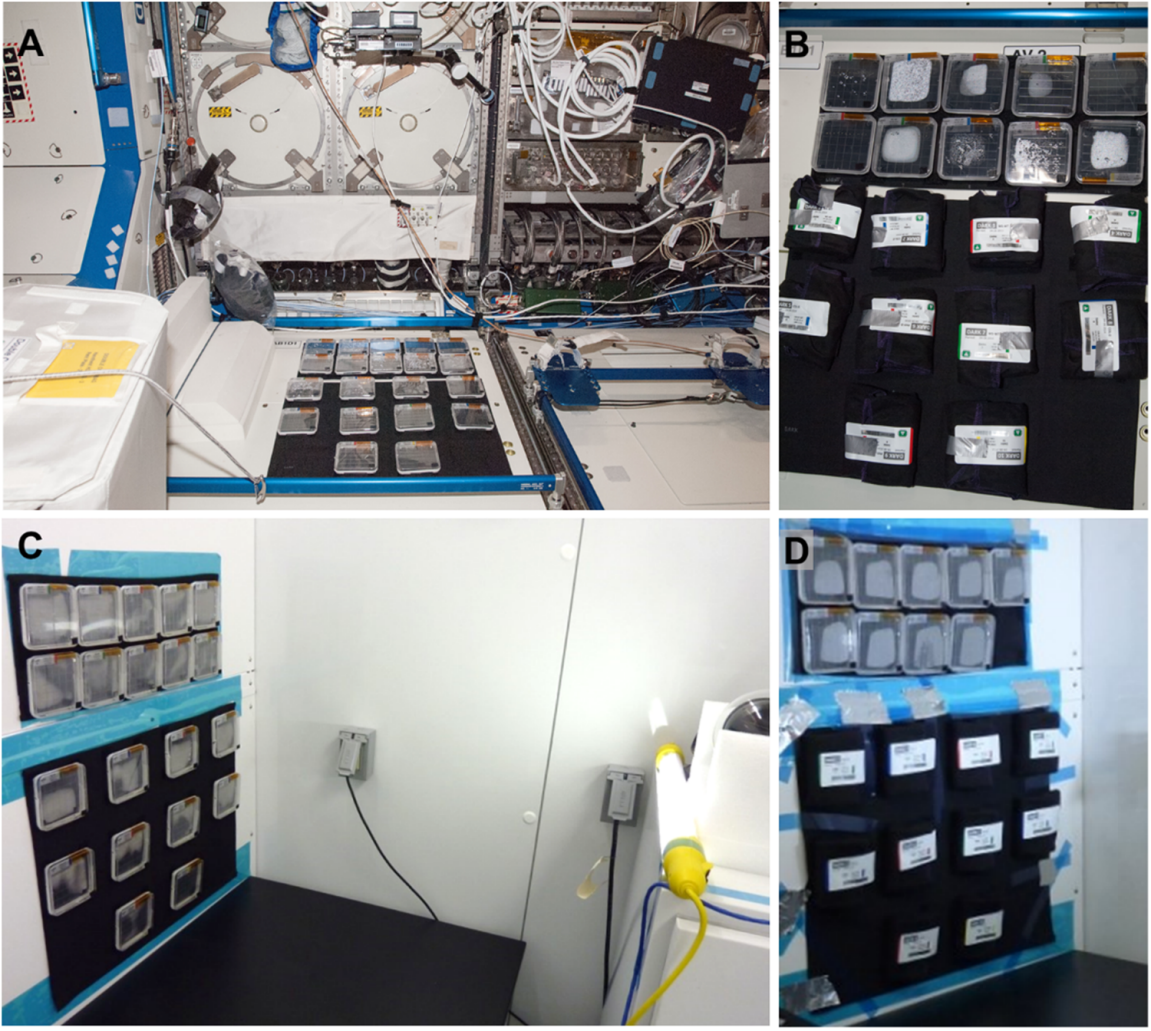
Experiment configuration and growth habitats. (A) The flight plates attached to the wall of the Destiny module of the International Space Station. (B) After germination was initiated by exposure to light, the dark-grown plates were re-wrapped in Duvetyne Black-Out cloth. (C) The ground control plates attached to the wall of the ISS Environment Simulations (ISSES) chamber at Kennedy Space Center in a vertical orientation for growth. (D) The dark-grown plates in the ISSES chamber after re-wrapping.

The dormant plates were activated on the ISS by bringing them out of cold stowage and removing the Black-Out cloth wrapping, 12 days after launch. The plates were then secured to a fabric support affixed to the wall in the US Lab of the Destiny module (Fig 1). The plants were allowed to grow on orbit for 11 days; some in the ambient light of ISS and some in the dark. A standard light meter was not available on the ISS, so ambient light levels were approximated through the use of iPad (iPad3, iOS 5.1.1) application “Light Meter HD- lux measurement tool”, which was executed by astronaut Steve Swanson. It was estimated that the light-grown plates received between 4–6 μmoles m^-2^ s^-1^ total light. The dark-grown plates were first activated by exposure to light, (the Black-Out cloth was removed from the designated plates for 4 hours), and then re-wrapped in Black-Out cloth for the duration of the growth period.

At 11 days, seedlings were photographed, and then harvested into Kennedy Space Center Fixation Tubes (KFTs) containing RNAlater. The plants were allowed to perfuse for 12–18 hours at ambient temperature within the KFTs, and then the KFTs were transferred to the MELFI (Minus Eighty-degree Laboratory Freezer for ISS). The frozen KFTs were returned to cold stowage within the Dragon Capsule at the end of the docked operations period, and were returned to Earth on May 18, 2014. Following the Dragon Capsule splashdown in the Pacific Ocean, the frozen KFTs were transferred to the Cold Stowage charter plane at the Long Beach Airport, placed into an insulated shipper with dry ice, and flown to Johnson Space Center (JSC). The KFTs were then transferred via FedEx ground to the Kennedy Space Center. The KFTs were removed from dry ice and transferred to a -80°C freezer on May 22, 2014. The PIs retrieved the plant samples from the KFTs on May 29, 2014 and transferred the frozen samples back to the University of Florida.

A comparable set of plates and operations comprised the experimental control set (referred to as the ground control). The plates of the ground control were grown at Kennedy Space Center (KSC) within the ISS Environment Simulator (ISSES) Chamber. The ground control was initiated 24 hours after the initiation of the spaceflight experiment to facilitate the programing the ISSES Chamber with the ISS environmental conditions experienced by the flight plates in the prior 24 hours. The environmental parameters that were monitored and then programed into the ISSES chamber included temperature, CO_2_ and relative humidity. In addition, the lighting in the ISSES chamber was adjusted to recapitulate the light levels being experienced by the plants on orbit. Light measurements in the ISSES chamber were taken with the same iPad model and operating system (iPad3, iOS 5.1.1) used on the ISS, with the “Light Meter HD- lux measurement tool” application.

### Sample preparation for transcriptomics

Seedlings preserved in RNAlater were removed from -80°C storage and then allowed to thaw at 4°C. The focus of the current study was restricted to the organ defined by the root tip, which was defined as the last 2mm of the root for the light-grown plants and the last 1mm for the dark-grown plants. The root tips were dissected from the intact plant roots while remaining submerged in RNAlater, and kept at or below 4°C until RNA extractions.

Three independent roots were used as biological replicates for the transcriptomic analysis. Dissected root tips were transferred into pre-labeled micro-centrifuge tubes, flash frozen in liquid nitrogen, and total RNA extracted using the Picopure RNA isolation kit (Arcturus®, Life technologies). RNA concentration was determined on a Qubit platform (Thermo Fisher/Life Technologies, Inc) and sample quality was assessed using the Agilent 2100 Bioanalyzer (Agilent Technologies, Inc.). Five ng RNA from each sample was used for RNA sequencing.

Sequencing experiments were performed at the Interdisciplinary Center for Biotechnology Research (ICBR) gene expression and sequencing core, University of Florida. Initial data analysis was performed at the ICBR bioinformatics core, University of Florida.

### Library preparation and Illumina sequencing

#### RNASeq libraries preparation using SMART-Seq V4 ultra low input RNA kit for sequencing combined with Illumina Nextera DNA Sample Preparation Kit

Five ng of total RNA was used for each cDNA library construction using the CloneTech SMART-Seq V4 ultra low input RNA kit for sequencing (Clontech Laboratories, Inc, cat#: 634890) according to the manufacturer's protocol. Briefly, first strand cDNA was primed by the SMART-Seq v4 oligonucleotide and then base-pairs with these additional nucleotides, creating an extended template. The reverse transcriptase then switched templates and continued transcribing to the end of the oligonucleotide, resulting in full-length cDNA that contains an anchor sequence, which served as a universal priming site for second strand synthesis. cDNA was amplified with primer II A for 9 PCR cycles. Then Illumina sequencing libraries were generated with 120 pg of cDNA using Illumina Nextera DNA Sample Preparation Kit (Cat#: FC-131-1024) according to manufacturer’s instructions. Briefly, 120 pg of cDNA was used as input for the Nextera tagmentation reaction (fragmentation and tagging) and then adapter sequences added onto template cDNA by PCR amplification. Libraries were quantitated by Bioanalyzer and qPCR (Kapa Biosystems, catalog number: KK4824). Finally, the libraries were pooled equal molar concentration and sequenced by Illumina 2X75 NextSeq 500.

#### NextSeq500 sequencing: RNA seq

In preparation for sequencing, barcoded libraries were sized on the Bioanalyzer, and quantified by QUBIT and qPCR (Kapa Biosystems, catalog number: KK4824). Individual samples were pooled equimolarly at 4 nM. This working pool was used as input in the NextSeq500 instrument sample preparation protocol (Illumina, Part # 15048776, Rev A). Typically, a 1.3 pM library concentration resulted in optimum clustering density (approximately 200,000 clusters per mm^2^). Samples were sequenced on three flowcells, using a 2x75 cycles (paired-end) configuration. A typical sequencing run in the NextSeq500 produced 750–800 million paired-end read with a Q30> = 85%. For RNA seq, around 40 million reads provided sufficient depth for transcriptome analysis.

#### Mapping reads to genome data, annotated transcripts, and profiling of gene expression

The paired end reads were mapped to the *Arabidopsis thaliana* var. Col-0 reference genome (TAIR10) using Spliced Transcripts Alignment to a Reference (STAR) software [35]. Multi-step process of transcriptome assembly and differential expression analysis was done using the Cufflinks tool [36]. Reads that map to each transcript were counted and normalized based on fragment length and total reads. Normalized counts were expressed in terms of FPKM values (fragments per kilobase of transcript per million mapped fragments). FPKM is directly proportional to abundance of the transcript. The expression data were generated at a False Discovery Rate = 0.05 (FDR). At this FDR, a total of 33,999 genes were tested.

### Cluster analyses and genome annotation

The cluster analyses used in all heatmaps were generated with GeneE (https://software.broadinstitute.org/GENE-E/). The clusters were generated using only genes that were assigned Atg numbers and called significant by the cuffdiff analysis (FDR 0.05), and were differentially expressed by at least two-fold (+/- 1 log2). For each cluster analysis, genes that showed significant differential expression in at least one comparison were sorted according to their Atg numbers and hierarchical clustering using Jaccard distance. Genes with a differential expression value of less than two-fold, and genes that have an FDR > 0.05 are depicted in gray. The fold-change color scale on the heatmaps ranges from -3 log2 (down 8-fold) to +3 log2 (up 8-fold). Genes with a differential expression value greater than 8-fold will be depicted with the same hue as for an 8-fold change. Gene annotations and functional relationships we obtained through ATTED-II (http://atted.jp/) [37].

### Availability of data

The datasets supporting the conclusions of this article are available in the Gene Expression Omnibus (GEO) repository, Series GSE94983 (https://www.ncbi.nlm.nih.gov/geo/query/acc.cgi?acc=GSE94983). In addition, the gene expression data and spaceflight-relevant metadata are deposited with NASA’s GeneLab repository (https://genelab-data.ndc.nasa.gov/genelab/accession/GLDS-120).

## Results

### Plant growth and development in the open ISS habitat

In contrast to the focused lighting and environmentally controlled habitat used in the APEX01 experiment [18, 38], the CARA Petri plates were deployed to a wall inside the Destiny module of the ISS, where they received just the ambient, low level, indirect lighting of the module. It was not possible to completely eliminate all lighting gradients, as the surface of the Petri plate mounted on the wall (both on the ISS and in the ISSES chamber for the ground controls) will naturally not receive ambient light through the surface flush to the wall. The plates also received the ambient cabin air, temperature and humidity (Fig 1A). The design of the experiment included a set of light-grown plates and dark-grown plates, with three replicate plates of each genotype (WS, Col-0 and *phyD*) in each condition. All of the plates were configured to keep the seeds in a dormant state until integration on the ISS, a process that included protecting the seeds from light with black cloth wrappings [34]. Thus, the first action of the astronaut was to expose all of the plates to light to activate germination (Fig 1A) and then to re-wrap the dark-grown plates in light-proof cloth (Fig 1B). Identical procedures were initiated in the comparable ground controls grown in the ISSES chamber (Fig 1C and 1D).

### Development without clear directional cues

**Fig 2:**
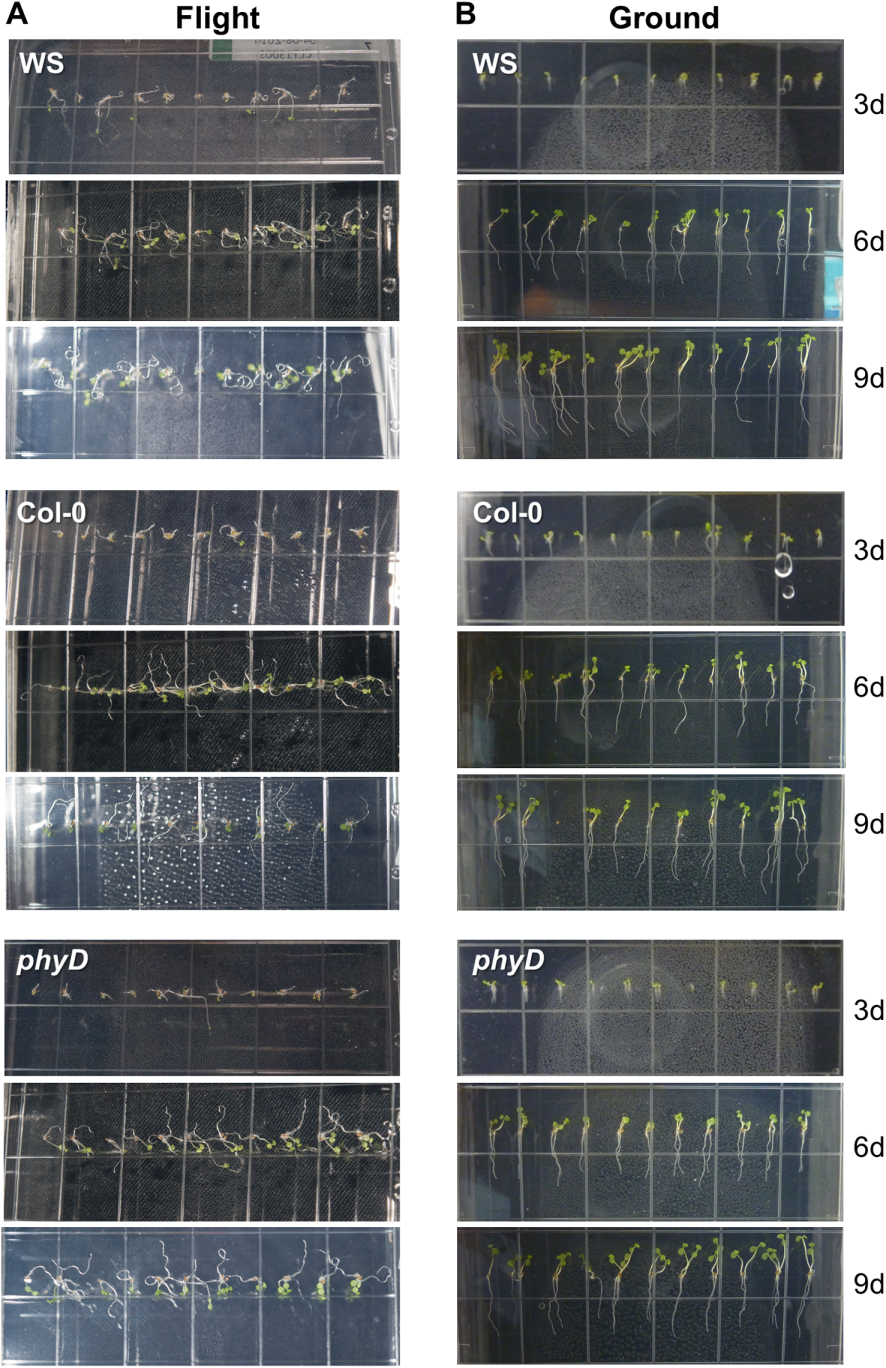
A comparison of the growth of the three genotypes in the light at 3 days, 6 days and 9 days. (A) The three panels in the left hand column show representative examples of Arabidopsis seedlings at 3, 6 and 9 days of growth on the ISS (Flight). (B) The three panels in the right hand column show representative examples of Arabidopsis seedlings at 3, 6 and 9 days of growth in the ISSES chamber for the ground controls (Ground). The images are taken through the back of the plate to minimize interference from condensation on the lid. The full set of uncropped Flight and Ground Control images can be found in the NASA GeneLab archives (https://genelab-data.ndc.nasa.gov/genelab/accession/GLDS-120).

The developmental progress of plant growth was followed through a series of photographs taken by the attending astronaut on the ISS, and by the attending technician at KSC, providing an independent “pseudonaut” on the ground in order to mimic astronaut activities on the ISS. The photographs in Fig 2 show representative images that compared the progress of growth after 3d, 6d and 9d for each genotype. The photographs were taken through the back of each plate. The region of interest was cropped from each astronaut-captured photograph to better display the progress of plant development. The full, uncropped, photographs are deposited in the NASA GeneLab database (https://genelab-data.ndc.nasa.gov/genelab/accession/GLDS-120). The spaceflight-grown plants are shown in the left-hand column (Flight).

In spaceflight, the diffuse light elicited root growth that was primarily perpendicular to the orientation of vegetative growth, in that the hypocotyls were growing away from the surface of the media, and the roots grew in coils along the surface of the solid media of the plate. This configuration of plant growth closely resembles patterns seen in plates grown horizontally on the ground (Phytagel surface parallel to Earth’s surface). However, unlike horizontal growth in a unit gravity environment, the root coils were loose and somewhat unorganized. In contrast, the roots of the ground control plants shown in the right-hand column (Ground) grew in a clear, positively gravitropic manner along the surface of the plate media. As described in the Methods section, the ground control plates were oriented vertically (Phytagel surface perpendicular to the Earth’s surface), a growth configuration that encourages root growth along the surface of the Phytagel in a reasonably straight, positively geotropic manner. The hypocotyls were generally oriented in a negatively geotropic manner, but were also angled away from the less well-lit surface of the plate adjacent to the wall, towards the relatively lighter environment of the room. These types of spaceflight growth patterns are distinctly different from what has been seen in the high-light plant growth hardware of Veggie [39] and the strong, directional lighting of the ABRS hardware [18, 19].

The developmental image series in Fig 2 shows that both the spaceflight (left column) and ground control (right column) plants of all three genotypes were virtually identical in terms of apparent health, vigor, and developmental age within each environment. However, due to the rather dim lighting environment, neither the light-grown spaceflight nor light-grown ground control plants had the developmental appearance Arabidopsis plants of comparable ages grown in the more optimal lighting regimes of the ABRS and Veggie plant growth habitats [18, 19, 39].

**Fig 3:**
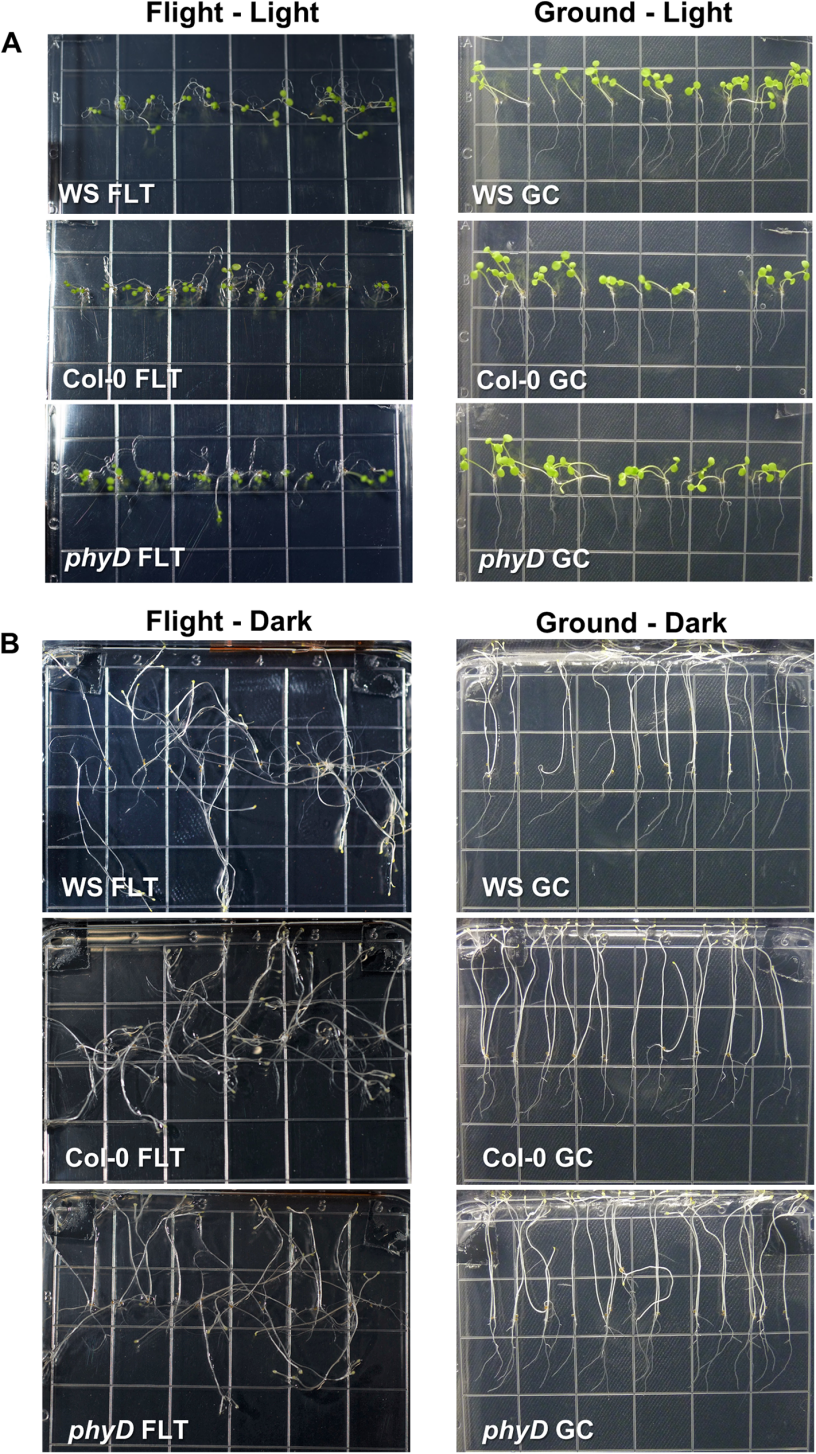
Growth and development of each genotype at the time of harvest. (A) The three panels in the left hand column show representative examples of seedlings just prior to harvest after 11 days of growth on the ISS in the light. The three panels in the right hand column show representative examples of seedlings just prior to harvest after 11 days of growth in the ground control ISSES chamber in the light. (B) The three panels in the left hand column show representative examples of seedlings just prior to harvest after 11 days of growth on the ISS in the dark. The three panels in the right hand column show representative examples of seedlings just prior to harvest after 11 days of growth in the ground control ISSES chamber in the dark. The full set of uncropped Flight and Ground control images can be found in the NASA LSDA archives (link TBD).

Final photographs of the plants were taken after 11 days of growth, and then the plants were harvested to KFTs (Kennedy Space Center Fixation Tubes) containing RNAlater. Fig 3 shows the photographs taken just prior to harvest. These photographs were taken from the front surface with the plate lid removed. The light-grown plants are shown in Fig 3A. The 11 day spaceflight seedlings (Flight) are shown on the left, and the comparable ground controls (Ground) are shown on the right. The dark-grown plants are shown in Fig 3B, again with the spaceflight grown plants on the left, and the ground controls on the right. There are some minor morphological differences in the manner in which the different genotypes grow, but these differences in coiling and skewing are well characterized features of each genotype, and consistent with morphometric evaluations from previous spaceflight experiments (e.g. [18, 40, 41]).

**Fig 4:**
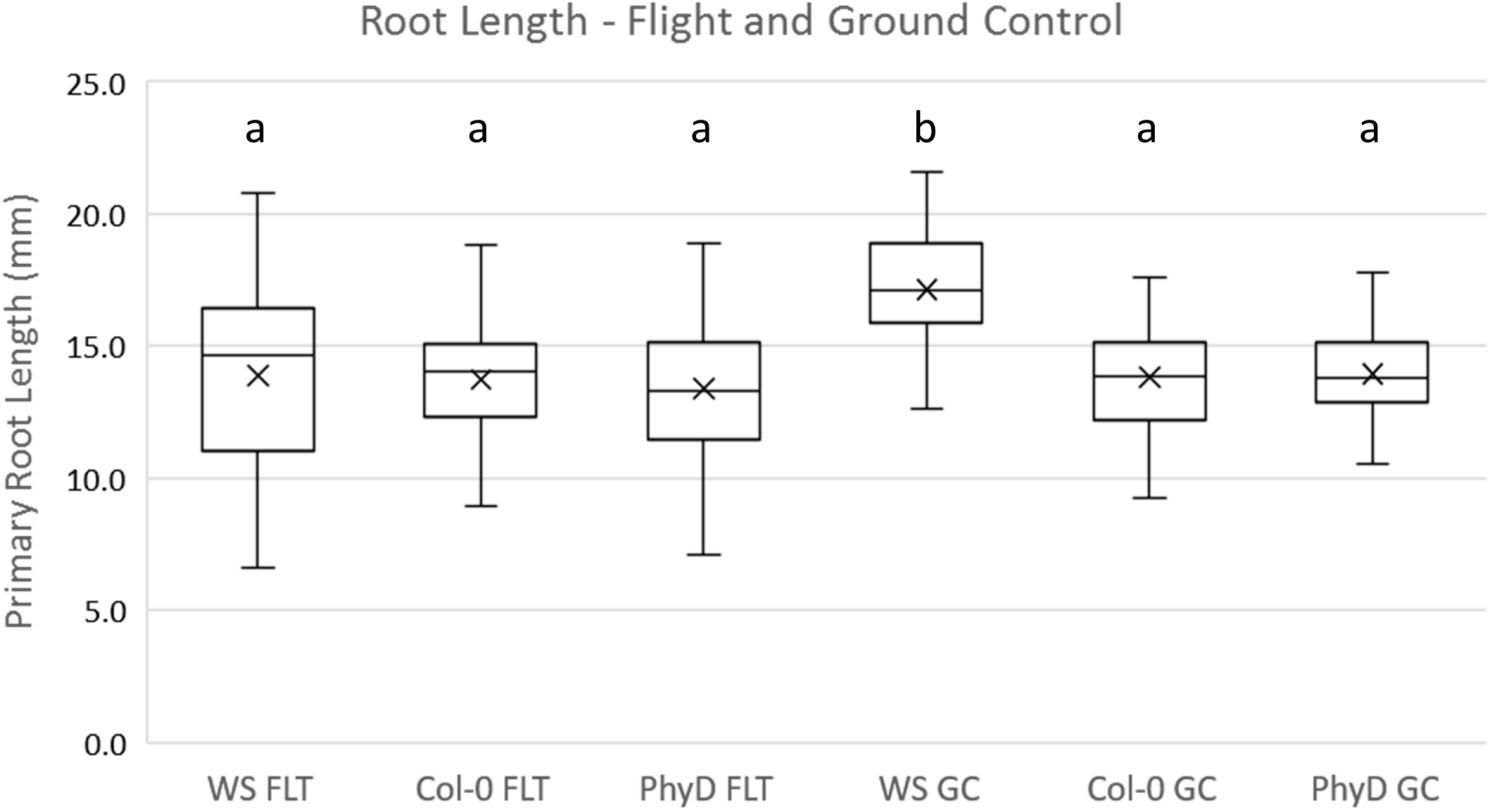
The root lengths among genotypes grown on orbit were not significantly different from one another. The box for each genotype/treatment combination contains a line indicating the median, and an "x" indicating the mean. Above each box is a designation of the results of Tukey’s Honestly Significant Difference, following the two-way ANOVA, where shared letters indicate insignificant difference. Only one of the six sample sets (WS.GC) was different from the other five.

All three genotypes were healthy on orbit as judged by appearance in the photographs of Fig 3, and all three genotypes were equally productive on orbit as determined by analysis of root length in Fig 4. These observations suggest that regardless of the gene expression changes among these genotypes, all three were equally able to adapt to spaceflight. Root length was chosen as a key, quantifiable trait for plant productivity in light-grown plants; root length has been associated with plant health and crop success [42], and the photos taken on the ISS provide clear resolution of root growth. Fig 4 compares the root length of each genotype in both spaceflight and ground control environments derived from the images. Root measurements were made using ImageJ, and the data were analyzed using R and two-way ANOVAs with Type II sum of squares to compare the independent variables of genotype and environment [40, 43]. When grown in the light, only WS grown on the ground was significantly different from any other combination of genotype and environment (F_2,310_ = 12.112, p<0.001), WS, Col-0 and *phyD* were not different from each other with regards to root length in spaceflight.

### Genotype matters in spaceflight transcriptional responses

**Fig 5:**
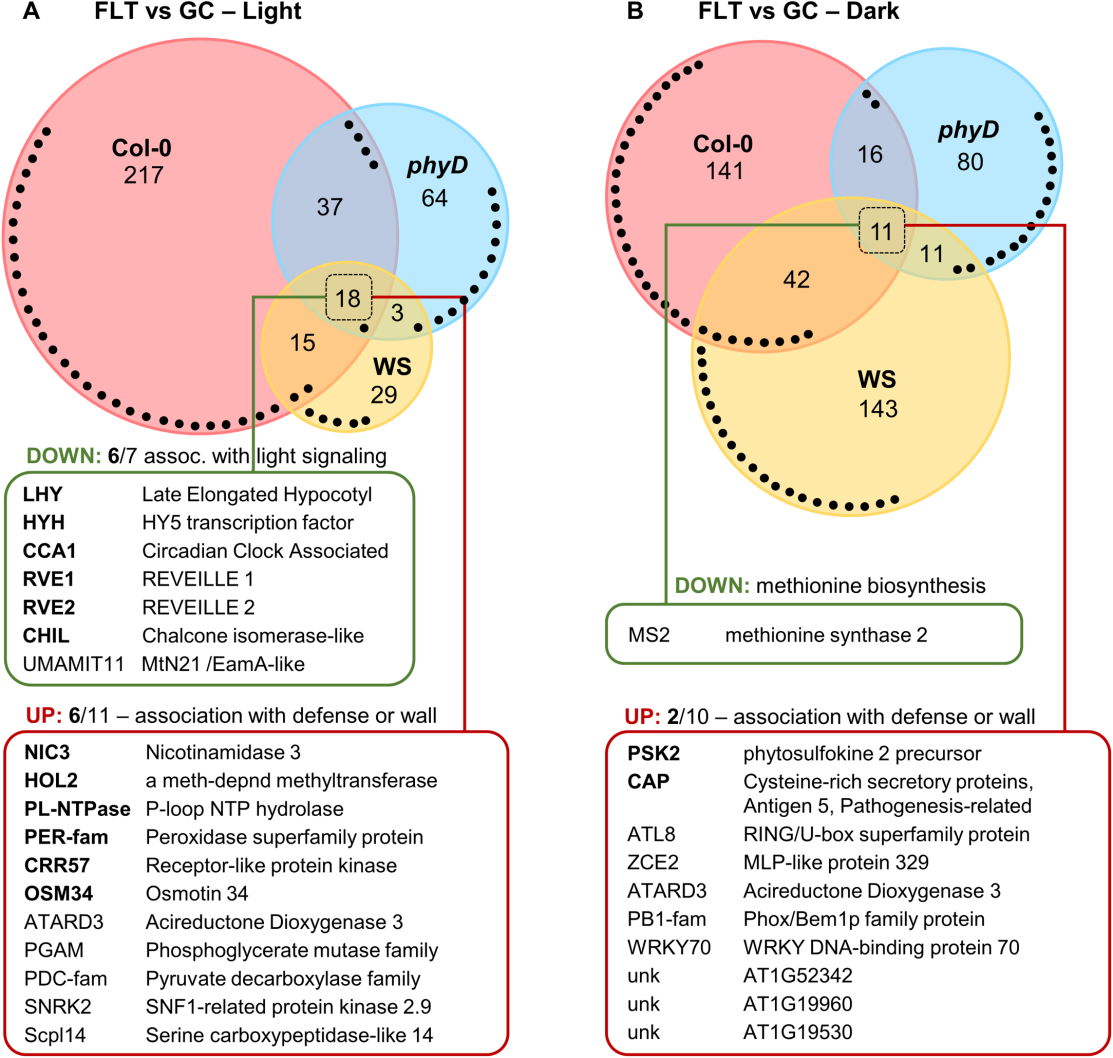
A Venn diagram displaying the distribution of coordinately expressed genes in response to spaceflight in all three genotypes. (A) Coordinately expressed genes in the root tips of light-grown plants. (B) Coordinately expressed genes in the root tips of dark-grown plants. Coordinately expressed genes common to all three genotypes are annotated below each diagram. The number of differentially expressed genes associated with cell wall processes are indicated as black dots within each representative sector of the diagram. The small number (11 in the light and 17 in the dark) of significantly expressed genes that are represented in two genotypes, but were changed in opposite directions (up in one genotype while down in the other), are not included in the enumerations of coordinately expressed genes in the Venn diagrams.

The transcriptional response that each genotype mounted in their respective environments was very distinct. The Venn diagrams of Fig 5 provide an overview of the Spaceflight to Ground root-tip transcriptome comparisons of each genotype; values from light-grown plants are shown in Fig 5A, and dark-grown plants in Fig 5B. The size of the Venn circles are to scale with respect to the numbers of differentially expressed genes. The genes that comprise the list show at least a two-fold change (-1<log2<1) with FDR<0.05. The greatest difference in the transcriptomes of spaceflight and ground control plants was seen in light-grown Col-0 plants, with a total of 297 genes. The spaceflight to ground differences in the other two light-grown genotypes were a total of 130 in *phyD*, and then only 71 in the WS genotype. A fourth (18/71) of the WS differentially expressed genes were also coordinately expressed in the other two genotypes. Almost all (6/7) of the coordinately expressed genes that were downregulated compared to the ground controls were associated with light signaling. In the upregulated genes, about half (6/11) were associated with plant defense or cell wall metabolism.

In the dark-grown plants, the distribution of differentially expressed genes between spaceflight and ground is quite different than was seen in the light-grown plants. In the dark, the WS and Col-0 cultivars showed roughly the same number of differentially expressed genes (218 and 224, respectively. In the dark, *phyD* exhibited the fewest differentially expressed genes (131). There were fewer coordinately expressed genes common to all genotypes (only 11). The single down regulated gene was associated with methionine biosynthesis, and two of the 10 upregulated genes have been associated with plant defense or cell wall metabolism.

Among the three genotypes, 129 genes were differentially expressed in both the light and the dark (Supporting S1 Table). However, only one gene was coordinately expressed in both the light and the dark in all three genotypes: ATARD3 (AT2G26400—Acireductone Dioxygenase 3). There were also a small number of genes that while overlapping in significant, differential expression between genotypes, rather than being coordinately expressed they were changed in the opposite direction. These genes (11 in the light and 17 in the dark) are not included among the genes represented in the Venn Diagram of Fig 5.

Genes associated with cell wall metabolism seemed prevalent among the differentially expressed genes. In order to create a standardized evaluation of the number of genes representing cell wall associated process, the differentially expressed gene sets used in Fig 5 were cross-referenced with two cell wall databases: the Purdue University Cell Wall Related Gene Families data sets (https://cellwall.genomics.purdue.edu/families/index.html) [44] and the SUBcellular localization database for Arabidopsis proteins (SUBA3—http://suba3.plantenergy.uwa.edu.au/) [45]. Genes were considered as cell wall associated if they were identified as such in either database. For graphic visualization, the numbers of genes identified with these comparisons are indicated as black dots within each Venn section in Fig 5. In addition, each gene ID number is identified with bold text and gold highlighting in the fully annotated Supplementary S2 and S3 Tables.

### Transcriptional responses between flight and ground

**Fig 6:**
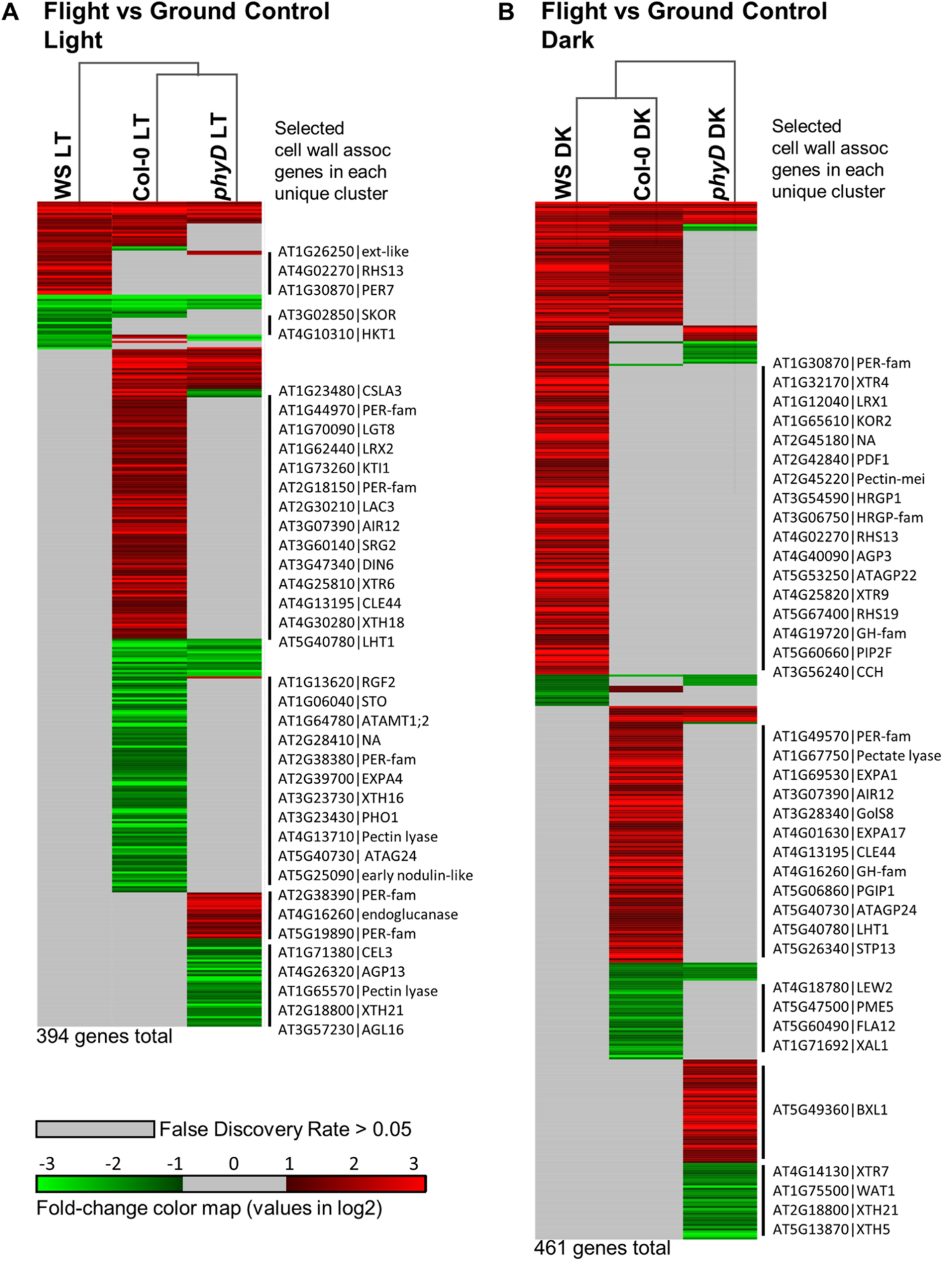
Arabidopsis root tip transcriptome heat maps showing differential expression between environments: Spaceflight compared to ground controls. The spaceflight to ground control heat maps in either the light (A) or the dark (B) are presented to the same scale. Red indicates upregulated and green indicates down regulated. Any gene indicated with a color is statistically significant (FDR<0.05) and expressed to a level of at least 2-fold (-1 <log2> 1). The color scale ranges from -3 log2 (down 8-fold) to +3 log2 (up 8-fold). Genes with a differential expression value greater than 8-fold are presented in the same hue as for an 8-fold change. Heat maps are annotated with selected cell wall associated genes that are unique to each genotype. The total number of genes represented in the heat maps are indicated below each map. The fully annotated heat maps are provided in Supporting S2 Table.

The genes enumerated in the Venn diagram of Fig 5 are shown as transcriptome heat-maps in Fig 6, in order to illustrate the distribution of differential expression among the genotypes along with fold-change. Fig 6 presents two sets of transcriptome heat maps that compare the patterns of gene expression between spaceflight and the equivalent ground controls for each genotype in the Light (Fig 6A) and in the Dark (Fig 6B). The heat maps were generated with GeneE software, utilizing statistically significant (FDR<0.05) expressed genes with differential expression of at least two-fold (-1<log2<1) in at least one comparison. The genes were sorted by Atg number, and hierarchical clustering was established using Jaccard distance. Each row of the heat map within each treatment (6A and 6B, respectively) corresponds to the same gene within each genotype. Significantly down regulated genes are indicated in green and significantly upregulated are in red. Genes depicted as gray did not meet either the fold-change or FDR criteria within that genotype. The gene rows are not collinear between 6A and 6B, but each heat map is presented to the same vertical scale. In the light, there were a total of 394 genes that were significantly, differentially expressed by at least two-fold in at least one genotype, and in the dark there were 461. Both the Light and Dark heat maps are annotated with the gene ID for selected cell wall-associated genes represented in the clusters of genes unique to a single genotype. The fully annotated heat maps can be found in Supporting S2 Table (FLT vs GC–Light) and Supporting S3 Table (FLT vs GC–Dark). In the Supporting tables, gene entries that have been associated with the cell wall (criteria described above) are indicated in bold text, and the gene ID number has been highlighted in gold.

In the light, the physiological adaptation of cultivar WS to the spaceflight environment required the fewest number of changes in gene expression, while cultivar Col-0 required about five times as many changes in gene expression. The mutation of the PhyD gene in Col-0 resulted in an almost 3-fold reduction in the number of differentially expressed genes in response to spaceflight. As presented in the Venn Diagrams of Fig 5, there were very few genes that were coordinately expressed among all three genotypes, and differentially expressed genes held in common between even two genotypes were also scarce.

The differentially expressed genes unique to each genotype were more abundant than those differentially expressed genes that were shared with another genotype. In the light, the upregulated differentially expressed genes unique to WS were largely associated with the cell wall, and processes in root hairs, including pathogen responses and development. The unique downregulated genes largely encoded transporters, along with several associated with plant defense, abiotic stress and cytokinin signaling. The over 200 uniquely expressed genes of Col-0 represented a wide range of diverse functions; defense, oxidative stress, cell wall metabolism, biotic and abiotic stress, and hormone signaling, and were well represented in both up and down regulated genes. The uniquely expressed genes of the Col-0 *phyD* mutant also encoded a similar diversity of gene categories, however *phyD* did not express the same representative genes from these categories, and there were fewer representatives of genes associated with stress responses in *phyD*. All three genotypes uniquely expressed about the same proportion of genes associated with the cell wall, with values ranging between 14% and 17%.

In the dark, WS and Col-0 exhibited about the same total number of differentially expressed genes for the physiological adaptation to spaceflight. About 20% were coordinately expressed in both cultivars, but the types of genes that are coordinately expressed do not appear to represent a substantially different category of genes than those which are uniquely expressed in each genotype. In the dark, *phyD* exhibited the smallest number of differentially expressed genes among the three genotypes, and only about 15% were also differentially expressed in Col-0. In the dark, although many genes were again associated with defense, oxidative stress, and cell wall metabolism, there were also more genes associated with hormone metabolism (auxin, ABA and GA), and gravitropism than were seen in the light. Again, all three genotypes uniquely expressed about the same proportion of genes associated with the cell wall, with values ranging between 14% and 16%.

**Fig 7:**
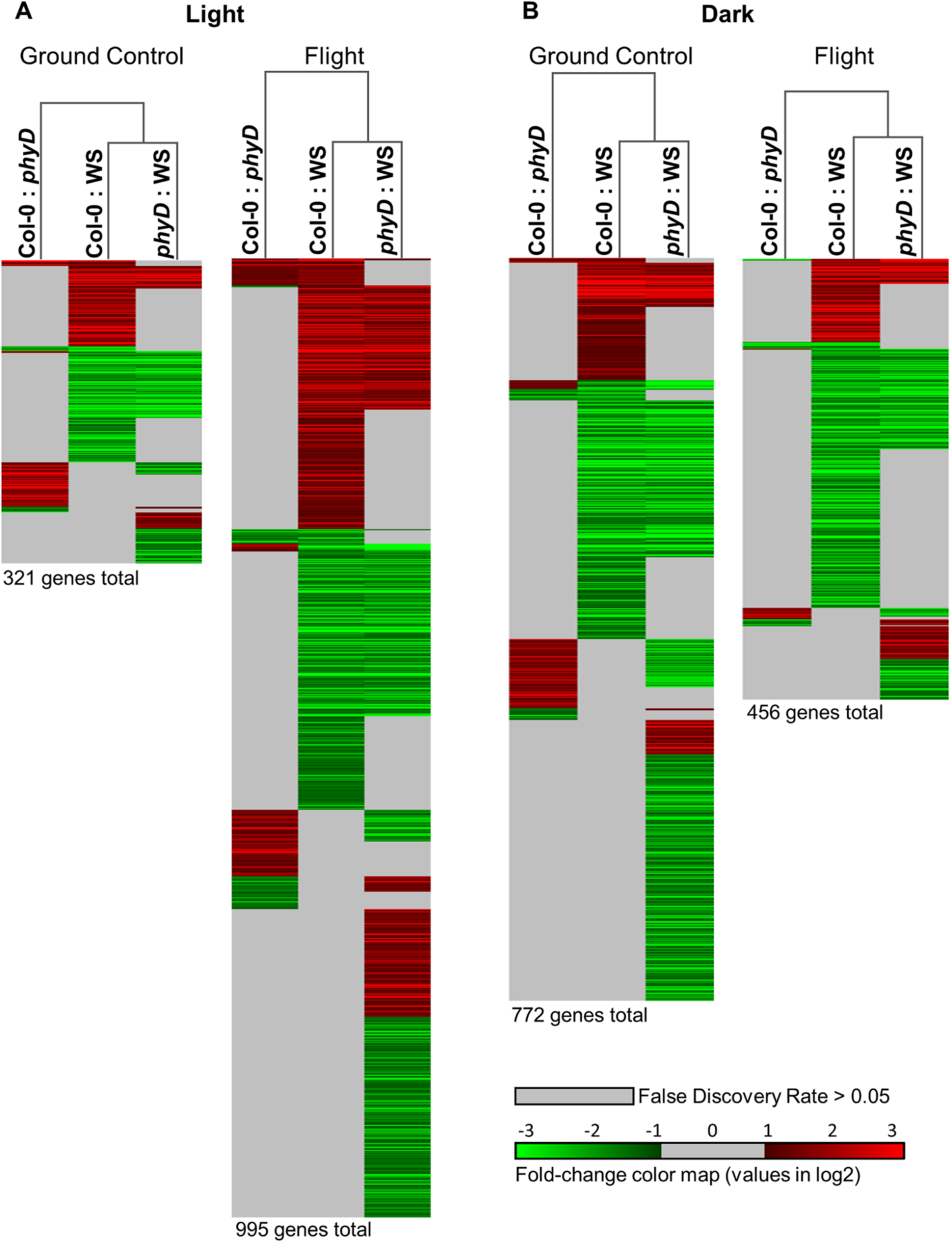
Arabidopsis root tip transcriptome heat maps showing differential expression between genotypes within each environment: Col-0 to WS, Col-0 to *PhyD*; then *PhyD* to WS. Each heat map is presented to the same scale. Red indicates upregulated and green indicates down regulated. Any gene indicated with a color is statistically significant (p<0.01) and expressed to a level of at least 2-fold (-1 <log2> 1). The color scale ranges from -3 log2 (down 8-fold) to +3 log2 (up 8-fold). Genes with a differential expression value greater than 8-fold are presented in the same hue as for an 8-fold change. The total number of genes represented in the heat maps are indicated below each map. The fully annotated heat maps are provided in Supporting S4, S5, S6, S7 and S8 Tables.

The transcriptomic differences exhibited by the different genotypes in response to spaceflight are underscored by the transcriptome differences between the genotypes in any given environment. Fig 7 shows the extent of how the transcriptomic responses of each genotype varied from each other when grown on the ground or in spaceflight. Pair-wise comparisons are shown between Col-0 and *phyD*, Col-0 and WS, and then *phyD* and WS; each within the four different environments: lighted environment on the ground, lighted environment in spaceflight, dark environment on the ground, and dark environment in spaceflight. In the light, there are fewer differences among genotypes in the ground controls than in spaceflight, whereas in the dark, there are more differences among genotypes on the ground than in spaceflight. The fully annotated heat maps can be found in Supporting S4, S5, S6, S7 and S8 Tables.

## Discussion

Plants in spaceflight differentially express genes compared to their ground controls, suggesting that physiological adaptation is taking place in plants on orbit. There have now been a number of transcriptomic and proteomic comparisons of Arabidopsis and other plants grown in spaceflight environments [19, 23, 38, 46–57] and the topic of plant growth in space has been recently reviewed [58–63]. In addition, there is more to growing in an orbital environment than the simple removal of gravity. Transcriptome comparisons of plants growing in artificial gravity environments generated by a centrifuge on the ISS illustrates that simply returning some form of gravity to the spaceflight environment does not completely ameliorate the transcriptional and proteomic response [51, 64, 65]. The general consensus from these studies is that while there is no single set of genes that are representative hallmarks of growth in the spaceflight environment, most plants do appear to differentially express genes associated with several consistent categories of genes. Genes associated with cell wall remodeling, cell polarity, and ROS signaling predominate, but the exact same genes are not utilized among all plants, plant organs, and experiments. Indeed there is no obvious consensus of what a canonical spaceflight response might be in plants or among plant cells, except perhaps for these categories of genes rather than specific genes.

Although root growth patterns were distinct between spaceflight and ground control plants, all three genotypes were developmentally comparable to the ground control plants. (Figs 2 and 3) and all three genotypes were equally productive in terms of root length in spaceflight. However, the molecular means to effect the physiological adaptation necessary to achieve comparable growth in the spaceflight environment was highly dependent on the genotype. Each genotype used almost completely different sets of genes to physiologically adapt their metabolism to the lighted environment of the ISS, and the number of differentially expressed genes was dramatically different among the genotypes. While Col-0 differentially expressed 297 genes to accomplish its physiological adaptation, WS accomplished adaptation with only 71 differentially expressed genes. Plants of the phyD mutant of Col-0 differentially expressed 127 genes. If differential gene expression is a measure of the degree or cost of the physiological adaptive response, then WS would be considered more highly spaceflight adapted than Col-0 and the *phyD* mutation would be considered a spaceflight adaptive mutation in Col-0 background. In other words, much of the Col-0 spaceflight response can be eliminated with a simple mutation in PhyD, without apparently compromising plant health or development or adding stress responses at the gene expression level. Indeed, the *phyD* plants did not appear to be at any disadvantage with respect to root navigation on orbit, where light is the primary tropic cue, nor did the *phyD* spaceflight transcriptome suggest a disruption of light-mediated signaling. Rather, relieving Col-0 of the gene encoding PhyD appeared to lighten the demands on the processes engaged to physiologically adapt to spaceflight. The converting the genome to WS further lightened the adaptive demands at the transcriptome level.

The data in Fig 5 further suggest that much of the differential gene expression exhibited by all three genotypes in the light of the ISS is non adaptive, or at least dispensable. For example, out of the 297 differentially expressed genes in Col-0, 217 of them appear dispensable, as neither *phyD* nor WS shared those differential gene expressions. Moreover, these three genotypes shared only 18 genes with similar differential gene expression patterns. This suggests the possibility that most of the gene expression responses in these genotypes, in these cells, were not fundamentally required for physiological adaptation of Arabidopsis to spaceflight.

The 18 coordinately expressed genes shared amongst the genotypes may well present two categories of adaptive metabolism that are universally important to growing in a spaceflight environment (Fig 5). The seven down-regulated genes in this set have all been associated with light signal transduction, whereas most of the eleven up-regulated genes have been associated with plant defense responses. These genes represent two strong trends seen throughout these data as well as other published transcriptomic responses of plants in spaceflight: modified light signal transduction and modified cell wall metabolism. Overall, the transcriptome responses for all three genotypes were enriched in these two categories, as well as for oxidative stress, genes associated with auxin, ABA and GA signaling, and general abiotic stress. The prevalence of representatives from these categories among the highly differentially expressed genes in all three genotypes could suggest that the adaptive process was generally the same for all three genotypes. However, the difference in the total numbers of differentially expressed genes among the genotypes could suggest that some genotypes expended far less effort to achieve a comparable adaptive metabolism.

Plants germinated and then grown in the dark on the ISS present a similar conclusion, although with different genes and differing numbers of gene expression changes. The *phyD* mutant differentially expressed fewer genes than Col-0, and only 11 genes were similarly differentially expressed among Col-0, *phyD* and WS. As was also seen in the light-grown plants, most of the differentially expressed genes of any particular genotype grown in the dark can be eliminated without affecting the ability to grow on the ISS.

It is important to note that the 18 genes shared among the differentially expressed genes of all three genotypes in the light of the ISS are almost completely distinct from the 11 shared differentially expressed genes of the dark grown ISS plants; only one gene, ATARD3, is common to both sets (Fig 5). ATARD3 has not been well characterized in the literature, but the gene has been loosely associated with pathogen response [66, 67] and cell wall metabolism [68]. Thus, in this experiment with three genotypes and two different ISS environments, there is only one universally differentially expressed gene in the root tips of spaceflight plants, and this gene is not represented in previously identified spaceflight transcriptomes. Therefore these data appear inconsistent with the concept that there is a basic required adaptive response to spaceflight that would be common among all plant cells and in under all conditions.

## Conclusions

The primary conclusion of this work is that much of the plant response to spaceflight is potentially non-adaptive in that at least most of the gene expression responses could be genetically eliminated without overtly compromising growth in space. This suggests that responses to truly novel environments may include responses from signals that are inappropriately activated or misinterpreted, and further suggests that these inappropriate responses can be genetically eliminated without adverse consequences. For spaceflight in particular, these data suggest that genetic manipulation can produce varieties that are better adapted to growth in spaceflight through elimination of unnecessary responses.

A secondary conclusion of this work is that there is no fundamental spaceflight response that characterizes plant physiological adaptation to spaceflight. Rather, the adaptive response is dependent upon the genotype of the plant and the specific environment within which it grows. The response also appears highly organ specific, as roots and leaves share few spaceflight differential genes in common [38, 47]. And yet, it remains possible and even likely that classes of differentially expressed genes will continue to be shared amongst cell types, genotypes and environments in space. However there is no overt indication that specific genes will be differentially expressed in all spaceflight plants.

These conclusions lie in sharp contrast to evolutionarily defined and well characterized terrestrial stress responses. Heat shock, cold shock, salt, pathogen attack and other stresses show commonality of differential expression across cell types and local environments. HSPs are HSPs in all plant cells. Since no such commonality exists in spaceflight responses, it appears that at least large portions of the spaceflight responses in plants are not necessary, and therefore can be eliminated without negative impact on plant life in spaceflight and opening the possibility of selecting plant genotypes optimized for spaceflight growth.

The differential expression of categories of genes, rather than individual genes, exhibited in the overall plant spaceflight response could be interpreted as support for the notion that certain categories of responses are either necessary for spaceflight physiological adaptation, or a common consequence of interpreting this novel environment. A common theme that appears to remain consistent among plant spaceflight transcriptomes is that of cell wall remodeling. Genes encoding proteins associated with cell wall remodeling have characterized the spaceflight response among several experiments and genotypes [23, 38, 47, 48, 51, 52, 56, 57, 65]. Cell wall remodeling genes are represented in the differentially expressed genes are richly represented in this experiment as well (Fig 6, Supporting S2 and S3 Tables). Thus cell wall remodeling appears to be, for the most part, a requirement or an unavoidable consequence of spaceflight physiological adaptation. Yet the differential gene expression that characterizes that response may be highly cell, genotype and environment specific, and indeed may not actually be required for successful adaptation.

Future experiments can be directed to establishing the minimal responses to spaceflight by further mutational and environmental manipulations, and can thereby contribute to systems engineering of spaceflight habitats. Plant growth in actual life support functions within space exploration is an important technical engineering challenge. Growing plants on petri plates allows what is likely the most benign growth system possible in spaceflight absent artificial gravity. Water management is not necessary, as the Phytagel provides water to the roots without potential flooding that can be seen in larger scale plant production systems on orbit or within exploration vehicles. Light and air management is quite similar to situations in terrestrial laboratories. It is quite possible that more stress responses will be seen in larger plant growth units on orbit, where water, nutrient and air management become more of an issue than for plants grown on plates. Engineering solutions to those issues can draw upon these plants-on-plates to better understand achievable stress reduction goals.

## Supporting information

S1 Table An annotated heat map table showing the differentially expressed genes in response to spaceflight represented in both the light and the dark grown plants. (XLSX)

S2 Table The fully annotated heat map displayed in Fig 6A: The spaceflight to ground control heat maps in the light grown plants. (XLSX)

S3 Table The fully annotated heat map displayed in Fig 6B: The spaceflight to ground control heat maps in the dark grown plants. (XLSX)

S4 Table The fully annotated heat map displayed in Fig 7A Ground Control: The transcriptome heat maps showing differential expression between genotypes for light grown ground control plants. (XLSX)

S5 Table The fully annotated heat map displayed in Fig 7A Flight: The transcriptome heat maps showing differential expression between genotypes for light grown spaceflight plants. (XLSX)

S6 Table The fully annotated heat map displayed in Fig 7B Ground Control: The transcriptome heat maps showing differential expression between genotypes for dark grown ground control plants. (XLSX)

S7 Table The fully annotated heat map displayed in Fig 7B Flight: The transcriptome heat maps showing differential expression between genotypes for dark grown spaceflight plants. (XLSX)

S8 Table A set of annotated heat maps summarizing differential expression between genotypes in all conditions: GC LT WS compared to GC LT Col-0, FLT LT Col-0 compared to GC LT Col-0, FLT LT WS compared to GC LT WS, FLT LT WS compared to FLT LT Col-0, GC DK WS compared to GC DK Col-0, FLT DK Col0 compared to GC DK Col0, FLT DK WS compared to GC DK WS, FLT DK WS compared to FLT DK Col-0, GC LT PhyD compared to GC LT Col-0, FLT LT PhyD compared to GC LT PhyD, FLT LT Col compared to GC LT Col-0, FLT LT PhyD compared to FLT LT Col-0, GC DK PhyD compared to GC DK Col-0, FLT DK PhyD compared to GC DK PhyD, FLT DK Col-0 compared to GC DK Col-0, FLT DK PhyD compared to FLT DK Col-0. Abbreviations: GC-Ground Control, FLT-Flight, LT-Light, DK-Dark
(XLSX)
